# Impregnation of Curcumin into a Biodegradable (Poly-lactic-co-glycolic acid, PLGA) Support, to Transfer Its Well Known In Vitro Effect to an In Vivo Prostate Cancer Model

**DOI:** 10.3390/nu11102312

**Published:** 2019-09-29

**Authors:** Eulalio Gracia, Andrea Mancini, Alessandro Colapietro, Cristina Mateo, Ignacio Gracia, Claudio Festuccia, Manuel Carmona

**Affiliations:** 1 Institute of Chemical and Environmental Technology (ITQUIMA), Department of Chemical Engineering, University of Castilla-La Mancha, 13071 Ciudad Real, Spain; Eulalio.Gracia@uclm.es (E.G.); Ignacio.Gracia@uclm.es (I.G.); 2 Laboratory of Radiobiology, Department of Biotechnological and Applied Clinical Sciences, University of L’Aquila, 67100 L’Aquila, Italy; mancio_1982@hotmail.com (A.M.); alecolapietro@gmail.com (A.C.); claudio.festuccia@univaq.it (C.F.); 3 Food Technology Lab, School of Architecture, Engineering and Design, Universidad Europea de Madrid, Villaviciosa de Odón, 28670 Madrid, Spain; cristina.mateo@universidadeuropea.es

**Keywords:** curcumin, PLGA polymer, supercritical carbon dioxide impregnation, curcumin release, prostate cancer, mice xenograft model

## Abstract

Prostate cancer (PCa) is one of the most common cancers in older men and is associated with high mortality. Despite advances in screening for early detection of PCa, a large proportion of patients continue to be diagnosed with metastatic disease, with ~20% of men showing a high tumor grade and stage. Medicinal plant extracts have a great potential to prevent/treat PCa, as well as to reduce its incidence/prevalence and improve survival rates. One of the most promising extracts is curcumin, which is a major, nontoxic, bioactive compound of *Curcuma longa*. Curcumin has strong antitumor activity *in vitro*. However, its potential beneficial in vivo affects are limited by its low intestinal absorption and rapid metabolism. In this study, curcumin was impregnated into a biodegradable poly(lactic-co-glycolic) acid (PLGA) support and characterized by FTIR and DSC, and its release by UV spectrophotometry. PLGA-curcumin was tested in different subcutaneous PCa xenograft models (PC3, 22rv1, and DU145 PCa cell-lines), and its effects evaluated by tumor progression an immuno-histochemical analysis (Trichromic, Ki67 and TUNEL stainings), were compared with those of a commercial curcumin preparation. Our results indicate that curcumin-impregnated PLGA is significantly more active (~2-fold increase) with respect to oral curcumin, which supports its use for subcutaneous administration.

## 1. Introduction

Prostate cancer (PCa) is the second largest cancer histotype and the fifth leading cause of cancer-associated deaths in older men worldwide [1,2]. There is evidence to suggest a close relationship between oxidative stress, inflammation, and risk of progressive PCa [3,4]. Oxidative stress, which is characterized by an imbalance between the production of reactive oxygen species (ROS) and the capacity of biological systems to counteract the effects of reactive free radicals or repair oxidative damage, plays a key role in PCa progression and its response to therapy [5,6]. Whereas low levels of intracellular ROS are critical for proper cellular signaling and homeostatic redox balance, high levels are deleterious and can lead to significant reductions in antioxidant defense mechanisms, which results in DNA, protein, and lipid damage [5,6,7]. Oxidative stress contributes to the initiation and progression of PCa by modulating transcription factors, cell cycle regulators, and DNA [5,8,9]. Against this background, several antioxidant-related phytochemicals have been tested for the prevention/treatment of PCa by ameliorating oxidative stress [10,11,12,13], and some have the added advantage of low toxicity [14]. Of the different families of compounds naturally present in plants, the polyphenols have shown the greatest activity against PCa. Some examples are silibinin, luteolin, ellagic acid, epigallocatechin gallate, and other catechins, or *trans*-resveratrol [15], which is widely found in food sources such as grapes, pomegranates, and green tea. Among these, the most promising compound tested for targeting PCa is curcumin. 

Curcumin has a long history of use as a food additive and traditional medicine in many Asian cultures. It has a broad range of biological activities, including anti-inflammatory, anti-oxidative, anti-metastatic, and multi-drug resistance reversing properties [16,17,18,19,20]. Curcumin is the major yellow pigment of turmeric (*Curcuma longa*), which is a spice found in curry powder. It functions by blocking cell signaling and inhibiting cell division through specific types of enzymes and growth factors that are directly involved in cancer development [21]. Pre-clinical studies have demonstrated the anti-cancer potential of curcumin via its effects on androgen receptor (AR) signaling and downstream targets (e.g., VEGF, PTEN, and NF-kB) [22,23,24,25]. Specifically, curcumin down-regulates AR expression [22], limits AR binding to the androgen response element of the prostate-specific antigen (PSA) gene, and reduces the expression of PSA in human prostatic LNCaP adenocarcinoma cells [26]. The United States Food and Drug Administration (FDA) has classified curcumin as being generally recognized as safe, and it is used as a supplement in several countries [27]. Moreover, phase I clinical studies show that oral curcumin is non-toxic to humans at doses up to 8000 mg/day for three months [28]. Nevertheless, there are several limitations to the use of curcumin as a therapeutic agent, including its poor bioavailability (low absorption, limited tissue distribution, and rapid metabolism) [29,30]. 

When ingested, curcumin is absorbed by passive diffusion through the plasma membrane of intestinal endothelial cells. It then undergoes numerous bio-transformations by the local action of bacteria and enzymes, and is recognized as a xenobiotic by specific transmembrane proteins belonging to the ABC-transporter family (e.g., P-gp, MRPs, and BCRP), which is responsible for translocating curcumin from the interior of the enterocyte to the lumen where it is eliminated by fecal excretion [31]. Accordingly, curcumin has low absorption and, thus, poor bioavailability.

The three most common strategies for overcoming the poor bioavailability of curcumin are: (1) concomitant consumption of curcumin with other natural compounds such as piperine [32], which inhibits the activity of ABC transporters and prevents the return of curcumin to the intestinal light [33], (2) chemical modification of the curcumin molecule to obtain analogues in the form of hydrazinocurcumin [34], diphenyl difluoroketone [35], or PAC (3,5-bis(4-hydroxy-3-methoxybenzylidene)-N-methyl-4-piperidone) [36], which are more soluble and bioavailable, or (3) encapsulation methods, for the design of drug delivery systems, such as nanoparticles or solid molecules of small dimensions (1 to 600 nm) [37], micelles formed by monolayers of phospholipids [38], liposomes with several layers of amphipathic lipids [39], or niosomes formed by surfactants or non-ionic surfactants that form vesicles without the presence of phospholipids and cholesterol [40]. 

Of all these options, the most attractive is encapsulation with biocompatible polymers. In this respect, poly(lactic-co-glycolic) acid (PLGA) is one of the most common biodegradable matrices used to encapsulate many compounds with biological activity, including the anti-tumor agents doxorubicin [41] and paclitaxel [42], and is approved by the FDA for contact with biological fluids, with potential applications in wound closure and surgical sutures, delivery carriers, tissue engineering scaffolds, or various types of implants [43,44].

In the case of curcumin, PLGA has been used to produce particles and nanoparticles using diverse techniques such as nanoprecipitation [45], coaxial electrospray processes [46], and liquid-driven co-flow focusing [47], or by methods that allow the particles to have super-paramagnetism [48]. In the present work, we used the supercritical carbon dioxide (scCO_2_) approach to fabricate a PLGA polymer impregnated with curcumin, which has been previously shown to result in the absence of residues and to have a high impregnation efficiency [49]. We characterized the in vitro release of curcumin and designed a protocol of periodic administration of this complex in three PCa xenograft models using PC3, DU145, and 22rv1 prostate carcinoma cell lines. We also compared this curcumin-impregnated PLGA scaffold with an improved commercial preparation (MicroActive© curcumin), which is designed to overcome the bioavailability difficulties. 

## 2. Materials and Methods 

### 2.1. Materials

For the production of the PLGA support, glycolide (G) and D,L-lactide (L) with a purity higher than 99.5% were purchased from Purac Biochem BV (Gorkum, The Netherlands). Curcumin used for impregnation had a purity higher than 98% (Wellgreen Technology Co. Ltd., Xi’an, China). MicroActive^®^ curcumin (25%) was supplied by Comercial Química Massó S.A. (Barcelona, Spain). Other reagents used were: CO_2_ (Carburos Metálicos, S.A., Madrid, Spain) with a purity of 99.5%, and stannous octoate (Sigma-Aldrich, Madrid, Spain), and acetone–analytical grade (VWR, S.A., Madrid, Spain). 

### 2.2. Impregnation of PLGA Using a Supercritical Carbon Dioxide 

Polymerization of PLGA in a molar ratio (L:G) 80:20 and the supercritical impregnation of polymer with curcumin was carried out as described [49], but with the following slight modifications: 1000 mg of solid PLGA was mixed with a solution of acetone containing 170 mg of curcumin and placed in a high-pressure vessel where CO_2_ was compressed to 150 bar at a temperature of 45 °C. The contact time for this impregnation was 8 h to ensure total solubilization. 

### 2.3. Impregnated Polymer Characterization

#### 2.3.1. Fourier-Transform Infrared Spectroscopy

Fourier-transform infrared (FTIR) spectra of the impregnated polymer were obtained with a Varian model 640-IR spectrometer (Varian Inc., Palo Alto, CA, USA) in the range from 4000 to 400 cm^−1^, with a resolution of 4.0 cm^−1^ and 64× scanning, using Varian Resolution software (Varian Inc.).

#### 2.3.2. Ultraviolet-Visible Spectrophotometry

Ultraviolet-visible spectrophotometry (UV-Vis) was used to determine the yield obtained in the PLGA impregnation step, as well as to establish the in vitro release of curcumin. In the first case, the high solubility of curcumin in acetone was used to calculate the yield. An eight-point calibration curve dissolving pure curcumin (0.025–0.250 mg/mL) in acetone was carried out (y = 7.664x − 0.0857, where y means concentration of curcumin in mg/ml and x absorbance reading, r^2^ = 0.999). For the in vitro release experiment, 30 mg of polymer impregnated with curcumin was suspended in 0.1 M phosphate buffered saline (PBS) (pH 7.4), in a 100-mL flask hermetically closed and protected from light, stirred at 100 rpm, and incubated in a shaking water bath at 37 °C. Samples of 5 mL were periodically removed from the flask to measure the quantity of curcumin released. The measurements were carried out using a Shimadzu UV-1603 dual beam UV spectrophotometer (Kyoto, Japan) at an absorption maximum of 421 nm using UVPC Personal Spectroscopy Software, version 3.6 (Shimadzu, Kyoto, Japan). 

#### 2.3.3. Differential Scanning Calorimetry (DSC)

Differential scanning calorimetry (DSC) analysis was determined using the DSC Q100 platform (TA Instruments, Newcastle, DE, USA). Samples of 3–10 mg were prepared in aluminum capsules. The samples were heated to 280 °C with a ramp of 10 °C/min, which is followed by cooling until −50 °C at the same rate and, lastly, heated again to 280 °C with the same ramp.

### 2.4. Xenograft Model 

Six-week-old male CD1 nude mice (Charles River, Milan, Italy) were maintained under the guidelines established by our Institution (University of L’Aquila, Medical School and Science and Technology School Board Regulations, complying with the Italian government regulation n.116, 27 January, 1992 for the use of laboratory animals). All mice received a subcutaneous flank injection of 1 × 10^6^ PC3, DU145 or 22v1 cells. Tumor growth was monitored by bi-weekly measurement of the tumor diameter using a Vernier caliper (length × width). Tumor weight (TW) was calculated according to the formula: TW (mg) = tumor volume (mm^3^) = d2 × D/2, where d and D are the shortest and longest diameters, respectively. The effects of the treatments were examined as previously described [50].

### 2.5. Treatments for In Vivo Experiments

**Figure 1:**
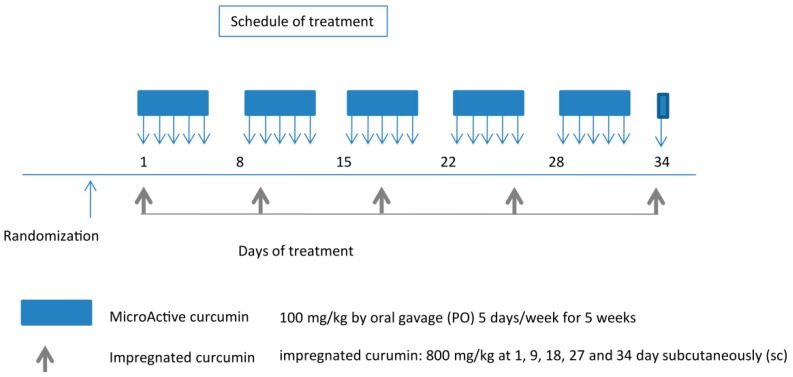
Protocol for the drug application to xenografted mice. MicroActive^®^ curcumin by oral administration (blue color), and impregnated curcumin by subcutaneous administration (grey color). Mice were randomized when tumors reached volumes of 0.8–1.3 cm^3^. At the end of the experiment (35 days after the start of treatments), animals were sacrificed by CO_2_ inhalation and tumors were removed surgically for analyses.

Before starting treatments, animals were randomized into three groups as follows: Group 1: mice (*n* = 10) receiving oral administration of 100 µL PBS (control). Group 2: mice (*n* = 10) receiving oral administration of 100 mg/kg MicroActive^®^ containing 25% curcumin (Maypro Industries LLC, Westchester, NY, USA) dissolved in methyl cellulose 0.5% five days weekly for five weeks. Group 3: mice (*n* = 10) receiving subcutaneous administration of PLGA-impregnated curcumin (800 mg/kg) inserted, as a powder, inside a subcutaneous dorsal pocket at day 1, 9, 18, 27, and 34. A scheme of the protocol followed is shown in Figure 1. Mice were randomized when tumors reached volumes of 0.8–1.3 cm^3^. The treatment performed on day 34 (one day before euthanasia) was done to maintain the selective pressure of the impregnated products. The injections of impregnated curcumin were performed distantly from the tumor cell inoculation sites. Accordingly, with cells injected placed in the animals’ flanks, the inoculations of the drug were carried out between the scapulae.

### 2.6. Evaluation of Treatment Response In Vivo

The following parameters were used to quantify the antitumor effects upon different treatments, as previously described [50]. (1) The tumor volume measured during and at the end of experiments. (2) The tumor weight measured at the end of experiment. (3) The tumor progression (TP or doubling time), defined as an increase of greater than 100% of tumor volume with respect to the baseline, and (4) time-to-progression (TTP), defined as the time for tumor progression. Taken together, these parameters provided the data on the percentage of tumors in progression to generate Kaplan-Meier curves.

### 2.7. Histopathology and Immunohistochemical Analyses

Indirect immunoperoxidase staining was performed on 4-μm-thick paraffin-embedded tissue sections. The Ki67 labeling index was determined by counting 500 cells at 100× magnification and determining the percentage of cells staining positively for Ki67. Apoptosis was measured as the percentage of TUNEL-positive cells measured in five random fields (400×) using the TACS® Blue Label kit (R&D Systems, Inc., Minneapolis, MN, USA). A consensus judgment was adopted [51] for immunohistochemical scoring of tumors based on the strength of positivity: negative (score 0), weak (score 1), moderate (score 2), or strong (score 3) staining. In each category, the percentage of positive cells was assessed by scoring at least 1000 cells in the area with the highest density of antigen-positive cells. The percentage of cells was graded as follows: 0 = absence of cells, 1 = <10% positive cells, 2 = positive cells in a range of 10–50%, and 3 = >50% positive cells. Overall expression was defined by the staining index (SI) and ranged between 0 and 9, with an SI≤4 indicating a low expression. Martius yellow, brilliant crystal scarlet, and soluble blue (trichrome) staining were used to stain erythrocytes, and, consequently, the presence of micro-thrombi, bleeding zones, and fibrous stroma.

### 2.8. Statistical Analysis 

Continuous variables were summarized as mean and standard deviation (SD) or 95% confidence intervals (CIs). Statistical comparisons between controls and treated groups were performed using analysis of variance or by the Student’s t test for unpaired data (for two comparisons). Dichotomous variables were summarized by absolute and/or relative frequencies. For dichotomous variables, statistical comparisons between control and treated groups were established by carrying out Fisher’s exact test. For multiple comparisons, the level of significance was corrected by multiplying the P-value by the number of comparisons performed (n), according to the Bonferroni correction. Overall survival was analyzed with Kaplan-Meier curves and the Gehan’s generalized Wilcoxon test. When more than two survival curves were compared, the Log rank test for the trend was used. This tests the probability that there is a trend in survival scores across the groups. All tests were two-sided and were determined by Monte Carlo significance testing. P-values of at least <0.05 were considered statistically significant. In the figures in which statistical analysis was performed, the significance is indicated by an asterisk. The SPSS (IBM Corp., Armonk, NY, USA) version 10.0 and StatDirect (version. 2.3.3., StatDirect Ltd., Altrincham, Manchester, UK) were used for statistical analyses and graphic presentations.

## 3. Results

### 3.1. Characterization of PLGA Impregnated with Curcumin in scCO_2_

**Figure 2:**
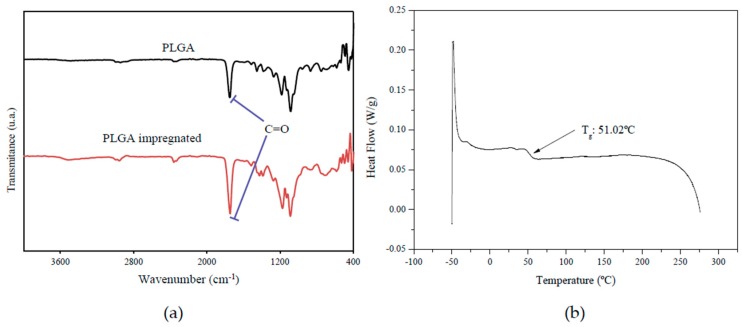
Characterization of the poly(lactic-co-glycolic) acid (PLGA) polymer alone and impregnated with curcumin in scCO_2_ by (**a**) FTIR and (**b**) DSC techniques.

The PLGA sample impregnated with curcumin in scCO_2_ is fabricated as described [49]. It was characterized using three different techniques. FTIR spectra showing different peaks corresponding to the PLGA-curcumin structure are represented in Figure 2a. The appearance of the most characteristic absorbance band of PLGA corresponding to the carbonyl group (1760 cm^−1^), which increased after impregnation due to the contribution of the C=O group of curcumin. This demonstrated that the process had taken place correctly. 

Curcumin-loaded PLGA samples were also characterized by DSC to determine the glass transition temperature (Tg), which is a useful measure to establish the purity of the resulting product and the level of residual contamination with the solvent used during the process. As shown in Figure 2b, the Tg of PLGA impregnated with curcumin was 51.02 °C.

Results of the UV-Vis spectrophotometry analysis against the calibration curve in acetone showed that, of the 170 mg of curcumin used for impregnation, 159.4 mg was actually impregnated, which resulted in an impregnation yield of 93.8%. 

### 3.2. Curcumin In Vitro Release

**Figure 3:**
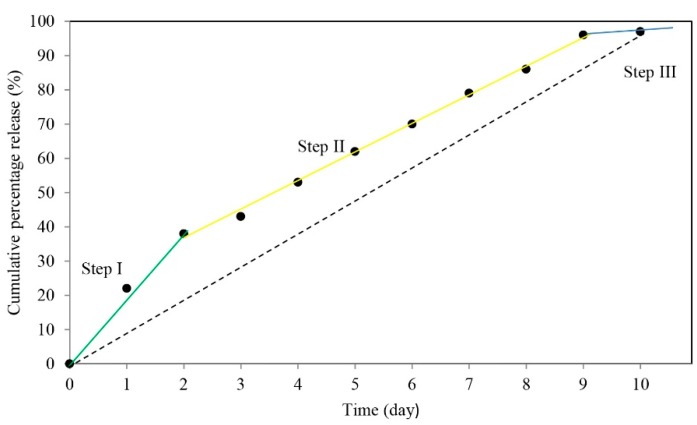
Curcumin release from impregnated PLGA with curcumin in scCO_2_ suspended in PBS at 37 °C for 10 days. Highlighted are the “burst” (step 1), “internal diffusion” (step 2), and “polymer degradation” (step 3) phases. See discussion for more details.

The amount of released curcumin was determined by UV-Vis spectra at 421 nm. A calibration curve in PBS was carried out to determine the release sample profile (Figure 3). 

According to the results shown in Figure 3, eight days were necessary for the release of more than 90% of the impregnated drug. After 10 days, almost all curcumin was released into the PBS solution. The previously determined content (159.4 mg of curcumin per 1000 mg of impregnated PLGA) and the results from the in vitro release analysis were used to design the animal model experiment. During the five weeks of the experiment, Group 2 (MicroActive^®^ curcumin) mice were treated five days weekly at the established concentration (100 mg/kg), as shown in Figure 1, until the day before euthanasia (day 34). This involved treating each mouse for 26 days with an equivalent amount of curcumin of 650 mg/kg (2600 mg/kg of MicroActive^®^ preparation containing 25% curcumin). Group 3 (impregnated curcumin) mice were treated five times over the five-week period, with a cadence of eight to nine days, which is the frequency established by the release trials. This involved treating the mice with a slightly lower amount of curcumin equivalent (636 mg/kg), since the 800 mg/kg dose on each occasion contained a lower proportion of curcumin (4000 mg/kg of PLGA impregnated with 15.9% of curcumin).

### 3.3. Antitumor Effect of Curcumin Preparations in PC3, DU145, and 22rv1 Subcutaneous Xenografts

**Figure 4:**
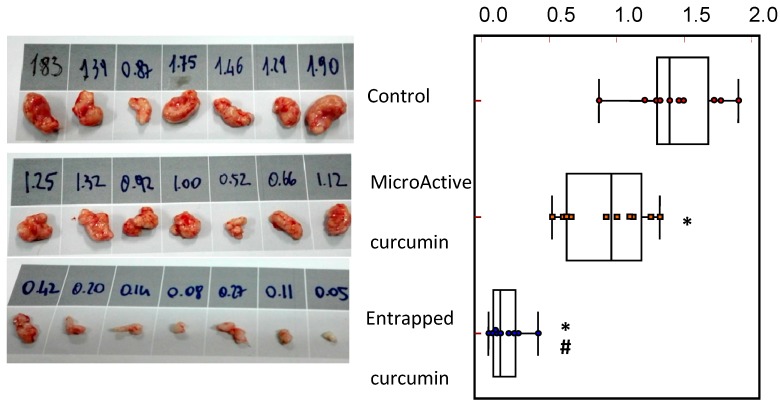
Aspect and weight of PC3 tumors (shown as an example for the analyses carried out for the three xenografts), evaluated at the end of the treatment period, and the statistical analysis by groups. * Statistically significant differences with the control group. ^#^ Statistically significant differences with a MicroActive^®^ group.

When compared with the control (PBS) group, both curcumin groups showed antitumor effects when administered in nude mice with PC3, DU145, and 22rv1 subcutaneous xenografts. In addition, both curcumin groups showed a significant decrease in tumor size, with the curcumin-impregnated PLGA group showing a significantly higher anti-tumor effectiveness when compared with the commercial MicroActive^®^ curcumin group. Figure 4 shows the changes in tumor weight by treatments, with a reduction of 29% in tumor weight in the MicroActive^®^ curcumin group and 71% in the curcumin-impregnated PLGA group.

**Figure 5:**
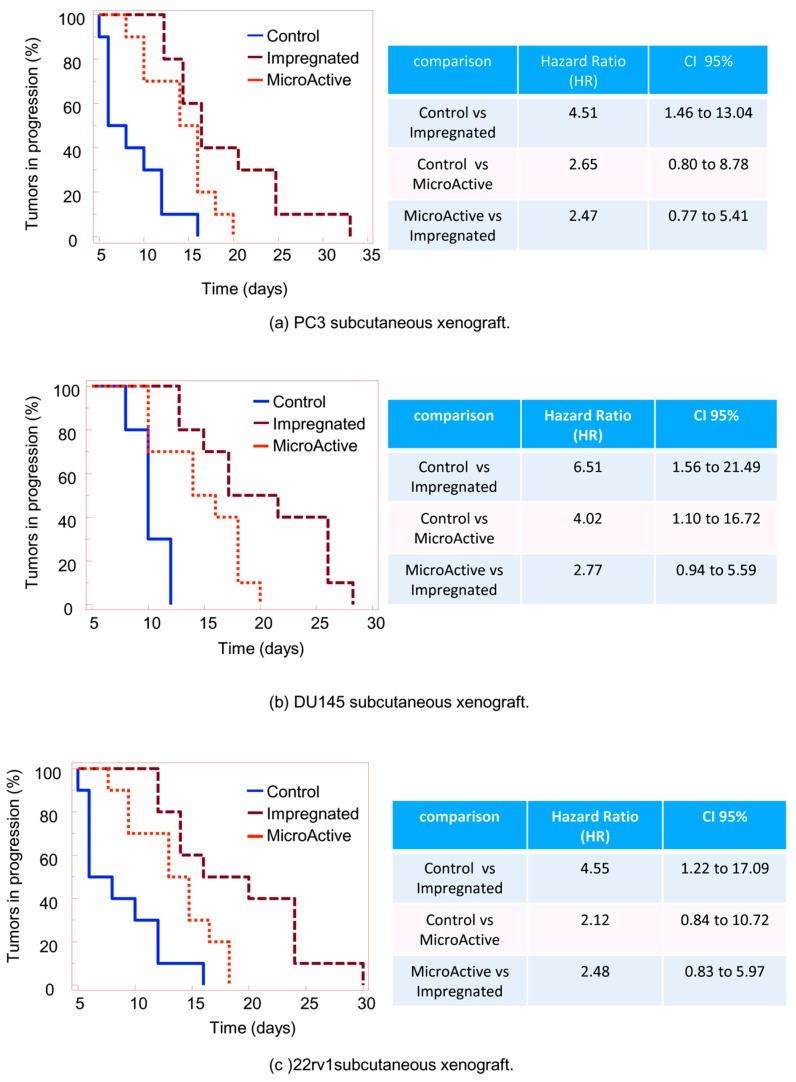
Tumor progression plotted over time using Kaplan–Meier analysis for (**a**) PC3, (**b**) DU145, and (**c**) 22rv1 xenografts. At the right side, statistical analyses of Hazard ratios of paired Kaplan-Meier curves.

**Figure 6:**
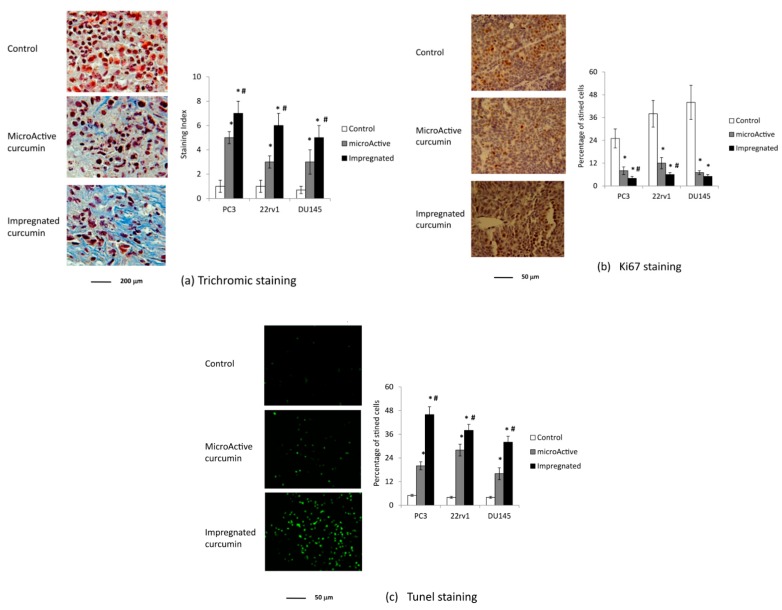
Histopathological/immunohistochemical analyses using three different staining techniques for tumor growth after PC3 cells subcutaneously injection in male nu/nu mice (xenograft model): (**a**) Trichrome staining, (**b**) Ki67 staining, (**c**) TUNEL staining, and statistical analysis for the three models of PCa xenografts. * Statistically significant differences with the control group. ^#^ Statistically significant differences with a MicroActive^®^ group. Magnifications are indicated in the single panels as size bars.

To reduce the probability of bias due to differences in tumor engraftment, we analyzed tumor progression using the TTP parameter, defined as the time (days) necessary to double the volume for each tumor, and comparing the differences in progression over time by the Kaplan-Meier analysis. As shown in Figure 5a, administration of curcumin-impregnated PLGA significantly reduced the progression of the PC3 tumors, with a Hazard ratio (HR) of 4.51 versus the control (CI95% 1.46 to 13.04), whereas the oral MicroActive^®^ curcumin had an HR value of 2.65 (CI95% 0.80 to 8.78). According to the performed Logrank test, a hypothesis test using a Kaplan-Meier estimator to compare the survival distributions of two samples, demonstrates that the survival rates (percentage of tumor progression) were statistically different in the considered comparisons (Figure 6), which the graphical inspection also demonstrates. However, the achieved HR best estimation showed, for some comparisons, a 95% CI including the unit. This could be due likely to the reduced statistical power (i.e., reduced sampling size). Administration of MicroActive or PLGA-impregnated curcumin preparations showed no side effects as indicated by the variations of body weight in treated animals versus controls (Table 1), while taking into account the contribution of tumors grown in animals.

### 3.4. Histopathology and Immunohistochemical Analyses

Histopathological and immunohistochemical analyses were used to confirm the greater effectiveness of curcumin-impregnated PLGA (Figure 6). Trichrome staining of tumor tissues from control and curcumin-treated animals showed that the deposition of collagen was higher in both curcumin-treated groups than in control animals (ranging from 20% to 50% of the slide area), with significantly stronger staining in the curcumin-impregnated PLGA group than in the oral curcumin preparation group (Figure 6a). This was consistent for all three cell-xenograft types. The increase in fibrosis was accompanied with a decrease in monocyte infiltration, which suggests reduced inflammation. Ki67 staining of tumor sections showed that both curcumin-treated groups had significantly less Ki67-positive cells than the control group (Figure 6b), with the curcumin-impregnated PLGA group showing significantly fewer Ki67-positive cells than the oral curcumin group for two of the three cell lines tested. Lastly, the percentage of TUNEL-positive cells (apoptotic, dead cells) was significantly greater in both curcumin-treated groups than in the control group, with significant differences between the curcumin-impregnated PLGA and the oral curcumin group (Figure 6c).

## 4. Discussion

### 4.1. Characterization of PLGA Impregnated with Curcumin in scCO_2_

Supercritical technology, specifically the use of scCO_2_ as a solvent, is a suitable medium for pharmaceutical applications due to its excellent properties [52]. This solvent also acts on the polymer matrix, allowing a phenomenon termed plasticization, which decreases the Tg of a polymer. This allows a better interaction between the pharmaceutical compound and the polymer [53]. This technology was applied in the present study for the copolymerization between lactide and glycolide (PLGA), which allows the fabrication of scaffolds with high porosity [54] and improves the drug loading capacity by providing more free space [49].

PLGA, which is FDA-approved, presents a wide variety of properties that make it a superb candidate for biomedical applications, such as the manufacture of tissue engineering scaffolds and delivery systems for different bioabsorbable medical implants [43,44]. The Tg reached by the impregnated polymer (51.02 °C) was high and very similar to that observed for the pure polymer (51.9 °C) [49], which indicates that acetone was completely removed from the polymeric matrix. This is an important feature for formulations to be used for prevention and treatment of cancer without any additional purification steps [55]. The impregnation yield was also high (93.8%), and superior to previous work using the same technique [49]. It is among the highest values ever achieved with PLGA used to encapsulate curcumin. This is above the 70% achieved using liquid-driven co-flow focusing (LDCF) [47], in the range of 89%–94% obtained by the single emulsion-solvent evaporation method [56]. This is close to the highest efficiency achieved so far, with 97.5% reported by Anand and colleagues [57], even though these authors obtained a very low percentage of drug loading (0.4%). This latter factor is also key when considering PLGA impregnation, since the higher the percentage, the lower the dose of the final product needed to administer the desired amount of the drug. In this sense, the results obtained in this work (15.9% drug loading) are well above the usual 5–10% [45,47], and just below the 30% reached using the electrospray coaxial technique [46]. 

### 4.2. Curcumin In Vitro Release

The mechanism for the controlled release of drugs from biocompatible polymers consists of three steps [7]: (1) solubilization of the drug located on the surface of the particles, which is the most accessible (burst stage), (2) diffusion of the drug through the pores (internal diffusion), and (3) solubilization of the entrapped drug when the polymer network is hydrolyzed (polymer degradation). Taking this drug delivery scheme as a reference, and in view of the results shown in Figure 3, the burst stage in our case was much less intense than in other PLGA-curcumin applications [45,47,56], likely because of the previously mentioned plasticizing effect, since there would be a better integration of curcumin with the polymer. The first stage over the first few hours is similar to the linear evolution that would be expected for the long-term release, whereas, in other systems, it is much more accentuated. For example, while in our study there is a release of ~20% in the first 24 h, the same percentage is reached in other studies within the first hour [56], which reaches 40% in the first 4 hours [45] or in the first day [47]. It seems that the effect of scCO_2_ for impregnation of curcumin allows a better homogeneous distribution of curcumin within the polymer matrix, which leaves only a small quantity accessible on the outside. This behavior is more characteristic of the release of a hydrophobic substance (such as curcumin) impregnated in PLGA, whereas the release of curcumin impregnated in PLGA by other techniques [45,47,56], is more similar to the behavior of a hydrophilic drug [7].

In summary, the high efficiency of impregnation, the high drug loading, and the better progressive release—without a burst stage—makes it an excellent choice in this application. In addition, it would allow for treatment of other pathologies beyond PCa, such as locoregional applications in unresectable cancers where intraperitoneal therapy may be a suitable delivery route. This approach has shown better prognosis and a longer survival rate for small tumors in patients with ovarian cancer [58]. The stable and continuous release would circumvent the toxicity normally associated with transferring approved intravenous protocols to an intraperitoneal bolus of the entire dose all-at-once [59], using, in this case, a biodegradable polymer that is well tolerated in locoregional applications in humans and is considered the polymer of choice for intraperitoneal treatments [60], as well as a drug that has an inherent low toxicity [28]. It would also be a suitable approach for solid pancreatic tumors, which are challenging to treat with chemotherapy because of their hypovascular and poorly perfused nature [61]. 

### 4.3. Antitumor Effect of Curcumin Preparations in PC3, DU145, and 22rv1 Subcutaneous Xenografts

Curcumin has high anticancer effects in in vitro tumor models and a relatively low anti-tumor effects *in vivo*. This could be due to its poor bioavailability, which restricts its application. In our study, we demonstrate that PLGA polymer impregnation with curcumin represents a good strategy to increase its effectiveness. We observed that impregnated curcumin shows higher anti-tumor effects over the oral curcumin preparation, which results in a ~2-fold increase in effectiveness when compared with MicroActive^®^ curcumin administration. We observed that animals remained healthy, as indicated by the relatively low body weight loss (Table 1). PLGA-impregnated curcumin preparation formed a foreign body granuloma-type lesion, which tends to disappear with the time concomitantly with the reabsorption of PLGA and release of curcumin. The more robust effectiveness was likely due to the higher concentration of curcumin in the tumor tissue, which would intensify the local anti-tumor effects as indicated by reduced Ki67-positivity and increased cell death. Apoptosis observed in several reports using curcumin was also associated with an increased fibrotic reaction in the treated tumor, as indicated by trichrome staining, as well as by reduced CD31 staining and vessel formation (not shown). We observed that impregnated curcumin does not appear to affect the health of the animals, as shown by the small body weight variation between groups (less than 10%, Table 1). We would conclude from this that there are no adverse side effects from the use of this curcumin preparation with regard to the doses released.

## 5. Conclusions

Curcumin is a natural anti-cancer compound that has been tested on a wide variety of human cancer cell lines and animal carcinogenesis models. Although chemotherapy is a cornerstone in the treatment of cancers in clinical practice, its efficacy is commonly restricted by an insufficient concentration of the drug at the malignant tissue and undesired side effects. While curcumin is regarded as an active anti-neoplastic agent, the major hurdle for its use as a chemotherapeutic agent is its low bioavailability, which limits its application. Several approaches have been developed to circumvent this problem without interfering with the anti-tumor effects of curcumin. 

PLGA polymer impregnation with curcumin seems to be an effective strategy to increase its effectiveness. The procedure for generating this type of polymer impregnated with curcumin is relatively simple and the conditions are well established, which should allow reproducibility and high impregnation yields to be achieved. This approach produces a slow release of curcumin release that could be theoretically maintained at a high level in the tumor for up to 10 days, with little lost by metabolism and intestinal uptake.

The distance between the site of injection of the tumor cells into the flanks and the subcutaneous dorsal application of the impregnated curcumin showed a systemic effect. The brief temporary adverse effects found around the dorsal pocket, suggest that the same compound could also be used in loco-regional approaches. In intraperitoneal applications or in the treatment of solid tumors such as pancreatic tumors, drug access is difficult due to its hypovascular and poorly perfused nature.

In summary, we report that curcumin-impregnated PLGA administered subcutaneously to PCa xenografts shows a clear and robust anti-tumor effect, with the bioavailability and controlled release of the curcumin likely responsible for an increase in anti-tumor responses compared with oral administration. PLGA particles are, thus, a potential delivery system that may hold promise for in vivo studies in other cancer types that might benefit from curcumin-related compounds.

## Figures and Tables

**Table 1:** Body weight comparison of mice, expressed in grams ± standard deviation.

Cell Line	Control *	MicroActive Curcumin *	Impregnated Curcumin *
PC3	25.5 ± 1.2	24.7 ± 1.8	24.0 ± 1.5
DU145	25.2 ± 1.4	24.8 ± 1.3	24.8 ± 1.3
22rv1	23.7 ± 1.5	21.9 ± 1.3	21.9 ± 1.3

* consider the contribution of tumors to the body weight.
